# Functional interleukin-17 receptor A is expressed in central nervous system glia and upregulated in experimental autoimmune encephalomyelitis

**DOI:** 10.1186/1742-2094-6-14

**Published:** 2009-04-28

**Authors:** Jayasri Das Sarma, Bogoljub Ciric, Ryan Marek, Sanjoy Sadhukhan, Michael L Caruso, Jasmine Shafagh, Denise C Fitzgerald, Kenneth S Shindler, AM Rostami

**Affiliations:** 1 Department of Neurology, Thomas Jefferson University, Philadelphia, PA 19107, USA; 2 Department of Ophthalmology, University of Pennsylvania, Scheie Eye Institute and FM Kirby Center for Molecular Ophthalmology, Philadelphia, PA 19104, USA; 3 Indian Institute of Science Education and Research-Kolkata (IISER-K), HC-VII, Sector-III, Salt Lake, Kolkata-700-106, India

## Abstract

**Background:**

Interleukin-17A (IL-17A) is the founding member of a novel family of inflammatory cytokines that plays a critical role in the pathogenesis of many autoimmune diseases, including multiple sclerosis (MS) and its animal model, experimental autoimmune encephalomyelitis (EAE). IL-17A signals through its receptor, IL-17RA, which is expressed in many peripheral tissues; however, expression of IL-17RA in the central nervous system (CNS) and its role in CNS inflammation are not well understood.

**Methods:**

EAE was induced in C57Bl/6 mice by immunization with myelin oligodendroglial glycoprotein. IL-17RA expression in the CNS was compared between control and EAE mice using RT-PCR, in situ hybridization, and immunohistochemistry. Cell-type specific expression was examined in isolated astrocytic and microglial cell cultures. Cytokine and chemokine production was measured in IL-17A treated cultures to evaluate the functional status of IL-17RA.

**Results:**

Here we report increased IL-17RA expression in the CNS of mice with EAE, and constitutive expression of functional IL-17RA in mouse CNS tissue. Specifically, astrocytes and microglia express IL-17RA *in vitro*, and IL-17A treatment induces biological responses in these cells, including significant upregulation of MCP-1, MCP-5, MIP-2 and KC chemokine secretion. Exogenous IL-17A does not significantly alter the expression of IL-17RA in glial cells, suggesting that upregulation of chemokines by glial cells is due to IL-17A signaling through constitutively expressed IL-17RA.

**Conclusion:**

IL-17RA expression is significantly increased in the CNS of mice with EAE compared to healthy mice, suggesting that IL-17RA signaling in glial cells can play an important role in autoimmune inflammation of the CNS and may be a potential pathway to target for therapeutic interventions.

## Background

IL-17A was described more than a decade ago [1], but became a major focus of research only recently, after a novel IL-17A-producing Th cell lineage (Th17) was discovered [2-6]. Th17 cells are generated in response to polarizing cytokines including TGFβ, IL-6, IL-23, IL-1β and TNF [7-10]. Like other inflammatory cytokines, IL-17A has both protective and pathogenic roles. IL-17A is important for host defense against infectious organisms [11-14]. However, elevated IL-17A in several autoimmune diseases including MS/EAE [15-17] contributes to disease pathogenesis. Deficiency or neutralization of IL-17A in EAE reduces disease susceptibility and clinical severity [18]. IL-17A can induce the expression of a range of inflammatory mediators, and thus modulates the activities of inflammatory cells [19,20] through production of numerous cytokines and chemokines involved in inflammatory responses [21].

Infiltration of inflammatory cells and encephalitogenic T cells in the CNS is the hallmark of EAE [22]. IL-17A expression is increased in lymphocytes derived from EAE mice, and anti-IL-17A antibody treatment during the recovery phase in a relapsing remitting EAE model delays the onset and reduces incidence and severity of relapses [23,24]. In MS patients, IL-17A mRNA and protein are increased in both brain lesions and mononuclear cells isolated from blood and cerebrospinal fluid [25,26]. Recently, it was demonstrated that IL-17A produced by Th17 cells is detectable at the blood brain barrier (BBB) in MS lesions, and that IL-17A can promote BBB disruption *in vitro *[27].

IL-17A functions through a distinct ligand-receptor signaling system [28]. IL-17RA is a widely expressed receptor that binds IL-17A with high affinity [29]. Leukocytes from mice lacking IL-17RA fail to bind IL-17A, and antibodies against IL-17RA inhibit the activity of IL-17A on human epithelial cells, indicating that IL-17RA is critical for IL-17A function [30]. Recently it has been demonstrated in infectious models in which neutrophils are crucial for host defense, that IL-17RA deficiency results in reduced chemokine levels and reduced neutrophil numbers, and increased susceptibility to infection [11,12]. IL-17RA signaling is implicated in both innate and adaptive elements of infectious and autoimmune diseases [15]; however, little is known about its signaling in the CNS. One reason may be that IL-17RA is expressed in the CNS at a very low level. Expression of IL-17RA in the CNS of healthy human subjects is undetectable by immunofluorescence but the receptor was expressed in CNS endothelial cells within heavily infiltrated MS lesions [27]. Given the important role that IL-17A plays in autoimmune diseases of the CNS, it is important to understand responses of CNS cells to IL-17RA signaling. Here, we have investigated expression and function of IL-17RA in healthy and inflamed mouse CNS tissues both *in vitro *and *in vivo*.

We report here that mouse CNS tissues express IL-17RA and the level of RNA expression increases in the CNS of mice with EAE. We also demonstrate *in vitro *that astrocytes and microglia in isolated culture express IL-17RA. The expression level of IL-17RA in microglia/macrophages is higher compared to astrocytes. Treatment of astrocyte cultures (devoid of microglia) and microglia cultures (devoid of astrocytes) with exogenous recombinant mouse IL-17A protein showed functional activation of IL-17RA signaling as demonstrated by increased chemokine secretion.

## Methods

### Mice

Eight-week-old and time pregnant C57Bl/6 mice were purchased from the Jackson Laboratory (Bar Harbor, ME). All animal procedures and care were conducted in accordance with approved ethical guidance under the auspices of the Thomas Jefferson University Animal Care and Use Committee. IL-17RA deficient mice on the C57Bl/6 background were used as negative control [12], and were kindly provided by David Abraham (Thomas Jefferson University, Philadelphia, USA) with permission from Amgen (Seattle, Washington, USA).

### Induction of EAE

Mice were injected subcutaneously with 100 μg myelin oligodendroglial glycoprotein (MOG_35–55_) peptide (MEVGWYRSPFSRVVHLYRNGK) in complete Freund's adjuvant containing 4 mg/ml *Mycobacterium tuberculosis *H37Ra (Difco, Michigan, USA) at two sites on the back. 200 ng pertussis toxin was given intraperitonially on day 0 and 2 post-immunization (p.i.). Mice were scored daily according to a 0–5 scale as follows: partial limp tail, 0.5; full limp tail, 1; limp tail and waddling gait, 1.5; paralysis of one hind limb, 2; paralysis of one hind limb and partial paralysis of the other hind limb, 2.5; paralysis of both hind limbs, 3; ascending paralysis, 3.5, = weakness of the upper limb, 4; moribund, 4.5; death, 5 [31]. At 20 days p.i. (peak of disease; average score 3) tissues were collected for mRNA extraction and histology.

### Histology

Mice were perfused transcardially with 40 ml of PBS followed by PBS containing 4% paraformaldehyde (PFA). Spleen, brain and spinal cord tissues were collected, post-fixed in 4% PFA overnight at room temperature (RT) and embedded in paraffin. 5 μm sections were processed and stained with Hematoxylin and eosin (H&E) for assessment of inflammation and Luxol Fast Blue (LFB) for demyelination. Sections were assessed as follows [31]; *Inflammation*: 0, none; 1, a few inflammatory cells; 2, organization of perivascular infiltrates; and 3, increasing severity of perivascular cuffing with extension into the adjacent tissue; *Demyelination*: 0, none; 1, rare foci; 2, a few areas of demyelination; 3, large (confluent) areas of demyelination.

### Immunohistochemical staining

Immunohistochemical staining was performed on mouse spleen, brain and spinal cord tissue sections by the avidin-biotin-immunoperoxidase technique as per manufacturer's instruction (Vector Laboratories, Burlington, California, USA) using biotinylated anti-mouse IL-17R antibody (R &D Systems, Inc.) directed against the IL-17RA extracellular domain and 3, 3' diaminobenzidine as substrate.

### Isolation of neonatal glial cell populations

Primary cultures of mixed glial cells from day 0 newborn mice were prepared as described previously [32]. Briefly, following the removal of meninges, brain tissues were minced with a Pasteur pipette and passed through 100 μm nylon mesh followed by a wash and centrifugation (300 × g for 10 min). The pellet was resuspended with a Pasteur pipette, passed through a 70 μm nylon mesh, followed by a second wash and centrifugation (300 × g for 10 min). Following dilutions with feeder medium {Dulbeco's essential medium (DMEM) containing 1% penicillin-streptomycin, 0.2 mM L-glutamine and 10% fetal calf serum (FCS)}, cells were plated and grown in a humidified incubator at 37°C. Cells were cultured until day 10, with a medium change on day 4, then every 2–3 days. To culture astrocytes free from microglia and to obtain pure microglial cultures, feeding of mixed glial cultures was stopped for the following 12–14 days. Cultures were then rigorously agitated for 30–40 min in an orbital incubator shaker at 200 rpm at 37°C to detach cells adhering to the astrocyte monolayer. Thereafter, cells suspended in the medium were collected and plated (8 × 10^5 ^cells/ml; 1.5 ml per chamber slide (Nunc, Rochester, New York, USA). After 15 min, non-adherent cells were discarded and adherent cells were maintained. Following this procedure, cells were 98–99% positive for CD11b (microglia/macrophage marker) and were negative for glial fibrillary acidic protein (GFAP), indicating a very high enrichment in microglia. Microglia were maintained in DMEM with 10% FCS, 1% Penicillin/Streptomycin and 1% L-glutamine. Adherent astrocyte monolayers from the original culture were trypsinized and resuspended in astrocyte specific medium at 8 × 10^5 ^cells/ml; and 2 ml were plated on each well of 6 well culture plates. Sub-cultured astrocytes were 85% positive for glial fibrillary acidic protein (GFAP) by immunofluorescence and 60–80% by flow cytometry. In a separate procedure, oligodendrocytes were isolated and enriched from neonatal mouse brain tissue as described earlier [33], producing cultures with 30–40% oligodendrocytes mixed with astrocytes.

### Immunofluorescence

Cells were processed by double label immunofluorescence for recognition of microglia and astrocytes. CD11b was used as microglia/macrophage surface marker; GFAP as an intracellular astrocytic marker; A2B5 as an oligodendrocyte precursor marker; and Galc as a mature oligodendrocyte marker. Unfixed cells were incubated with biotinylated anti-CD11b primary antibody for 30 min at RT followed by Cy3 conjugated streptavidin secondary antibody for 30 min. Cultures were then rinsed with Ham's F12, fixed in 95% ethanol 5% acetic acid (vol/vol) at -20°C for 10 min and washed in Ham's F12 (Invitrogen). For GFAP staining, cells were washed 3 times with PBS, followed by PBS with 0.5% Triton X-100 and PBS with 0.5% Triton X-100 and 2% heat-inactivated goat serum. Cells were incubated with polyclonal GFAP antisera (DAKO, Carpinteria, California, USA) for 30 min, washed, and labeled with Cy2-conjugated goat anti-rabbit IgG. Cells were then washed, mounted into Mowiol, and visualized by fluorescence microscopy (Olympus I X-80) with a 20 PlanApo oil immersion objective (1.0 numerical aperture). Images were acquired with a SensiCam^QE ^High Performance CCD Camera.

For the detection of IL-17RA protein expression on the cell surface of isolated astrocytes and microglia, unfixed cells were incubated with anti-IL-17R (S-18. sc-1902; Santa Cruz Biotechnology, Inc. Santa Cruz, California, USA) primary antibody for 30 min at RT followed by Cy2-conjugated Hamster anti-goat IgG secondary antibody for 30 min. Cultures were then rinsed with Ham's F12, fixed in 2% PFA for 10 min and washed in Ham's F12, mounted and visualized by fluorescent microscopy as mentioned above. Double label immunofluorescence was performed by using anti- GFAP mouse monoclonal antibody raised in mouse in combination with anti-IL-17R raised in rabbit. For microglia cultures we used biotinylated anti-CD11b primary antibody in combination with Cy3 conjugated streptavidin secondary antibody and anti-IL-17R.

### Flow cytometry

Glial cell cultures were harvested and washed in buffer containing 1% FCS, 0.1% NaN_3 _in PBS, and stained with an APC-conjugated antibody to CD11b for 20 min in the dark at 4°C. Cells were washed, fixed and permeabilized using Fix and Perm^® ^cell permebilization reagents (Caltag Laboratories, Burlingame, CA). Cells were then stained for intracellular GFAP with polyclonal anti-GFAP antibody and PE-conjugated goat anti-rabbit IgG. Polyclonal anti-mouse IL-17R-Carboxyfluorescein (R&D Systems) was used to quantitatively determine the density of IL-17R on the cell surface by flow cytometry. Cells were FC-blocked by treatment with 1 μg of mouse IgG/10^6 ^cells for 15 min at RT prior to staining. Protein G-purified normal goat-IgG conjugated with carboxyfluorescein (CSF) (R&D Systems) was used as isotype control for IL-17RA staining.

### IL-17A treatment in vitro

Functional studies were performed either on confluent microglia subcultures obtained after 24 hr, or astrocyte subcultures obtained after 72 hr hrs of plating. On the day of stimulation, media were removed and cells were washed with PBS. Recombinant mouse IL-17A (10 ng/ml or 1–100 ng/ml where indicated) was added to the selected culture wells. Non-stimulated sister cultures were used as controls throughout the studies. Culture supernatants were collected at 3, 6, 12, 24 and 48 hr time points.

### Search light chemokine arrays

Levels of 29 analytes including cytokines, chemokines, growth factors and matrix metalloproteinases (Table 1) in supernatants of cultures either treated with IL-17A or non-stimulated, were assayed using a SearchLight Multiplex Sandwich ELISA according to the manufacturer's instructions.

**Table 1:** List of analytes including cytokines, chemokines, growth factor and matrix metalloproteinases measured by multiplex array

**Cytokines**	**Chemokines**	**Growth Factor**
GM-CSF	KC	TGFβ
IFN-γ	MCP-1	**Matrix** **Metalloproteinases**
IL-1α	MCP-5	
IL-1β	MIP-1α	
IL-2	MIP-1β	
IL-4	MIP-2	MMP-2
IL-5	MIP-3β	MMP-3
IL-6	RANTES	MMP-9
IL-10		
IL-12p40		
IL-13		
IL-17		
IL-18		
IL-23		
IL-27		
TNF-α		

### Extraction of RNA and synthesis of cDNA

Tissue RNA and cellular RNA was extracted with RNeasy Midi or Mini kits (Qiagen, Chatsworth, CA) respectively according to the manufacturers' recommendations. The purity of total RNA was assessed using a NanoDrop^® ^ND-100 spectrophotometer (NanoDrop Technologies, Wilmington, DE). One μg of total RNA was used to synthesize cDNA with high capacity cDNA archive kit (Applied Biosystems Inc., Foster, CA) according to the manufacturers' instructions.

### Real time PCR

Quantitative Real-Time (RT)-PCR was performed on the ABI PRISM 7000 Sequence Detection System using TaqMan^® ^Universal PCR Master Mix (Applied Biosystems) and TaqMan^® ^Gene Expression Assays primer/probe (Applied Biosystems; Assay ID: Mm00434214_m1-from exon boundary 1–2) according to the manufacturer's specifications. Additional primer probe was also selected from exon boundary 3–4 (Assay ID. Mm01183143_m1) for amplification as this region is disrupted in IL-17RA deficient mice. To generate a standard curve for quantification of templates, cDNA constructs either from exon boundary 1–2 or 3–4 were cloned into pGEM^® ^T Easy vector (Promega, Madison, Wisconsin, USA) and verified by double strand sequencing. Respective cDNA constructs were serially diluted 7 times at a ratio of 1:10. Thus, the dynamic range for each gene was from 12 to 12,000,000 copies. Samples were analyzed in triplicate and experiments performed three times. Amplification data were analyzed with ABI Prism Sequence Detection Software 2.1 (Applied Biosystems).

### Statistics

2-tailed, Student's Welch corrected t tests (for parametric data) were used for statistical analysis. Differences were considered significant if * p < 0.05.

## Results

### IL-17RA is constitutively expressed in CNS tissues

**Figure 1:**
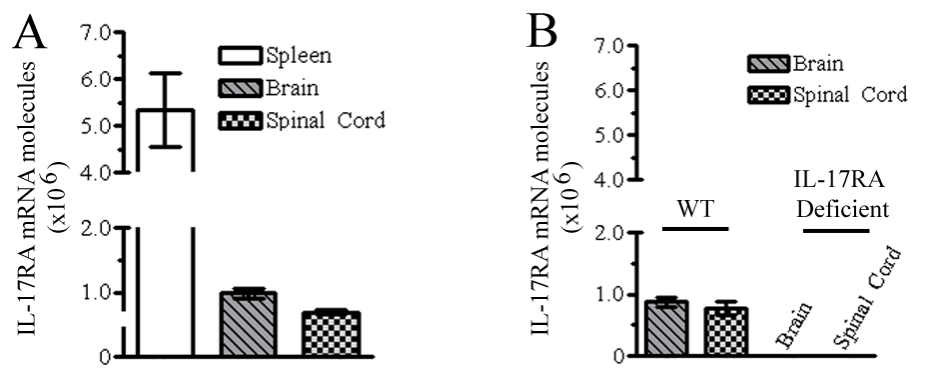
**IL-17RA expression in mouse CNS**. Spleen, brain and spinal cord were harvested from 10-week-old C57BL/6 and IL-17RA-deficient mice and used for quantification of IL-17RA mRNA by RT-PCR. Absolute copy number (mRNA molecules/μg total RNA) is shown. (A) *Quantification of IL-17RA mRNA in wild-type mice using a primer set from exon boundary 1–2*. IL-17RA expression was detected in all samples with > 5-fold more expression in spleen than CNS. One experiment of three is shown. (B) *Expression of IL-17RA assessed by RT-PCR using a primer set from exon boundary 3–4*. IL-17RA expression was again observed in wild-type (WT) CNS, but not in IL-17RA-deficient mice. Mean and SEM generated from multiple animals in one experiment (of three) is shown.

IL-17RA is expressed in most tissues examined to date, although little is known about its expression in the CNS. To investigate if IL-17RA is expressed in normal CNS tissues, we harvested brain, spinal cord and, as a positive control, spleen, from 10-week-old C57BL/6 female mice. RNA was extracted and cDNA synthesized for quantitative RT-PCR. Pearson's correlation coefficient of the standard curve generated from serially-diluted cDNA constructs of pGEMT-IL-17RA exon boundary 1–2 was 0.99. IL-17RA was expressed in both brain and spinal cord with slightly higher levels detected in brain. Levels of IL-17RA mRNA in normal CNS were approximately 5-fold lower than that of normal spleen (Figure 1A). Spleen has previously been shown to have the highest levels of IL-RA expression [29], and CNS expression was found to be slightly higher than heart, skeletal muscle, and testes, the tissues with the lowest expression levels [29]. To reconfirm the expression of IL-17RA in CNS, we constructed another standard curve using a plasmid expressing the IL-17RA gene from exon boundary 3 – 4, and used IL-17RA deficient mice (in which IL-17RA gene is disrupted between exon boundary 4 – 11) [12] as a negative control. Pearson's correlation coefficient of the standard curve generated from serially-diluted cDNA constructs of pGEMT-IL-17RA exon boundary 3–4 was 0.98. No detectable amplification was observed in samples from IL-17RA deficient mice, while IL-17RA mRNA was again detected in brain and spinal cord of wild-type C57BL/6 mice (Figure 1B). These results demonstrate that normal mouse CNS tissues constitutively express IL-17RA.

### IL-17RA expression is upregulated in inflamed CNS

**Figure 2:**
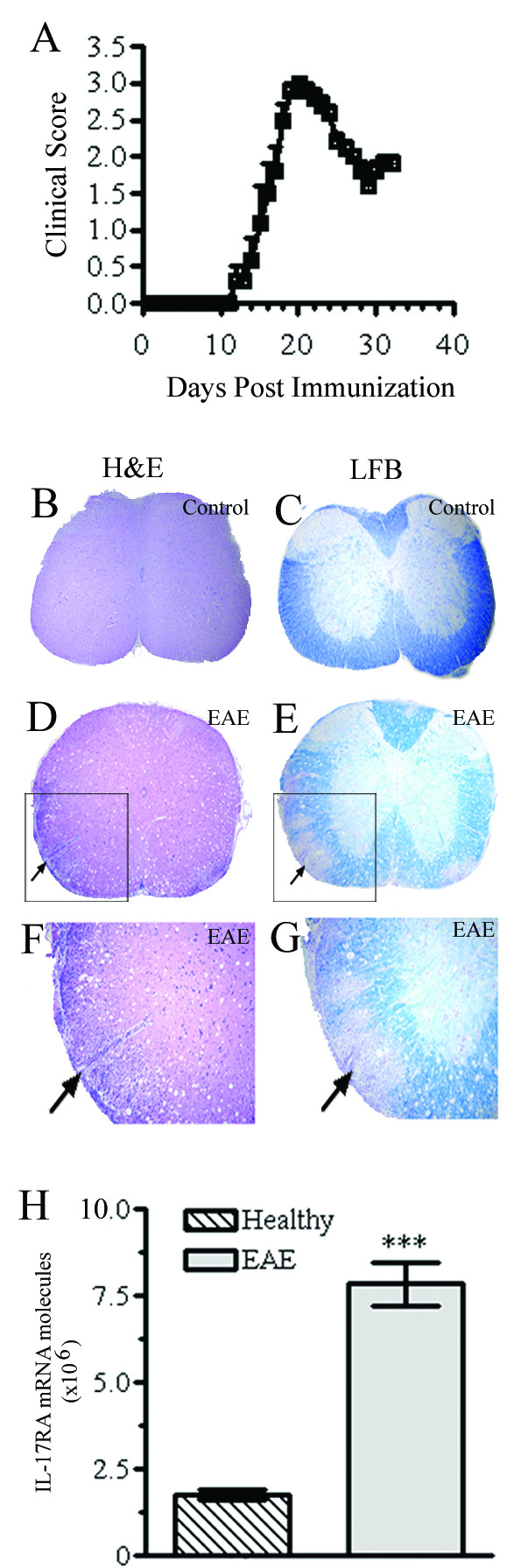
**IL-17RA expression in the CNS of EAE mice**. (A) *Clinical profile of EAE*. Female C57BL/6 mice (n = 8) were immunized with MOG_35–55 _and scored daily. Data represent mean clinical scores ± SEM. One experiment of three is shown. (B-G). *CNS inflammation and demyelination*. Mice were sacrificed at day 20 p.i., spinal cords were harvested and 5 μm sections were stained with H&E (B, D, F) or LFB (myelin stain; C, E, G). Magnifications are 40× (B-E) and 100× (F, G). EAE mice had significant cellular infiltration (arrows; D, F) and demyelination (arrows; E, G). No inflammation or demyelination occurred in control mice (B, C). (H) *IL-17RA expression is up-regulated in the inflamed CNS of EAE mice*. EAE and control mice (n = 5) were sacrificed at day 20 p.i. and IL-17RA expression from isolated spinal cords was assessed by RT-PCR using a primer set from exon boundary 1–2. Expression of IL-17RA in EAE mice is upregulated > 5-fold (*** P < 0.0001).

Mounting evidence suggests that IL-17A causes pathology in autoimmunity, but little is known about mechanisms of IL-17RA signaling. To examine if CNS inflammation alters IL-17RA expression locally, we utilized the EAE model induced in C57BL/6 mice with MOG_35–55_. As shown in Figure 2A, these mice developed the classical clinical profile of EAE. Spinal cords were harvested at the peak of disease (day 20) for histopathological studies and RNA extraction. In agreement with clinical findings, we observed inflammatory demyelinating lesions in EAE mice (Figure 2D–G). Quantitative RT-PCR using a standard curve (expressing the gene from the exon boundary 1 – 2) demonstrated nearly 5-fold more IL-17RA expression in EAE spinal cords than healthy controls (Figure 2H). These results suggest that inflamed CNS may have heightened responsiveness to IL-17A.

### *In situ *detection of IL-17RA protein

**Figure 3:**
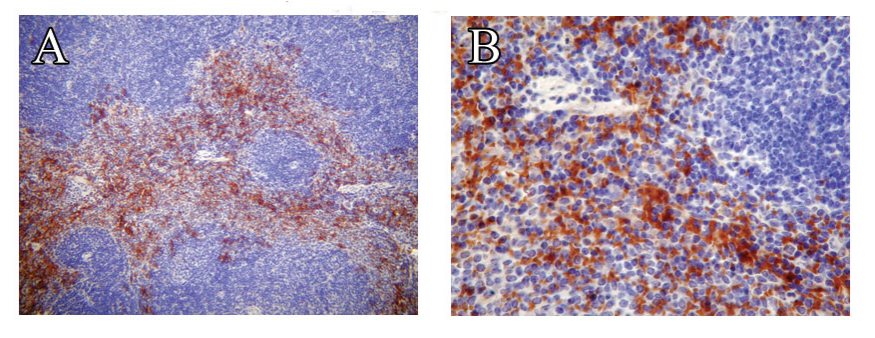
**Expression of IL-17RA protein in spleen tissue from adult mouse**. 5 μm thick serial cross sections of adult mouse spleen were processed for immunohistochemisty and immunostained with anti-IL-17RA antibody (A, B) followed by hematoxyline counterstaining. Immunostained sections demonstrated a subpopulation of IL-17RA positive cells (appear in brown). Original magnification for A was 100× and B was 400×.

**Figure 4:**
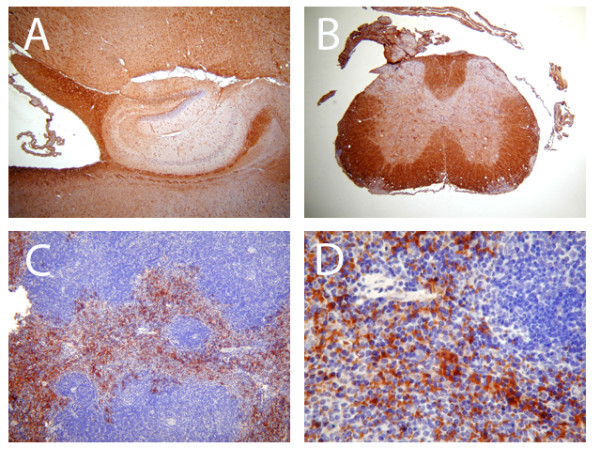
**Expression of IL-17RA protein in brain, spinal cord and spleen tissue from adult mouse**. 5 μm thick serial cross sections were processed for immunohistochemisty. Immunostaining with anti-IL-17RA antibody and counterstaining with hematoxyline demonstrated a subpopulation of IL-17RA positive cells in spleen cells as shown here and in Figure 3 (C, D; appear in brown). Immunostained section of brain surrounding the hippocampal region (A) and cross section of spinal cord (B) demonstrated non-specific binding of IL-17RA. Original magnification for A, B and C was 100× and D was 400×.

To investigate *in vivo *cell specific expression of IL-17RA at the protein levels in healthy mouse spleen (positive controls) and CNS tissue we performed immunohistochemistry. Immunohistochemical studies showed that in spleen a subset of cells, more specifically matured splenocytes surrounding the germinal center, express IL-17RA protein (Figure 3A and 3B) and cells within the germinal center showed very little, if any, staining. In CNS tissues, high background staining with 3, 3' diaminobenzidine made interpretation difficult and unreliable (Figure 4). We have performed western blot from the tissue lysates of spleen, brain and spinal cord tissues using commercially available antibody from R & D. None of the antibody was compatible for mouse tissue lysates. Because *in vivo *CNS cell-specific detection of IL-17RA in healthy or EAE mice was uninterpretable, *in vitro *cultures of specific CNS cell types were used to further examine IL-17RA expression.

### Glial cells express IL-17RA

**Figure 5:**
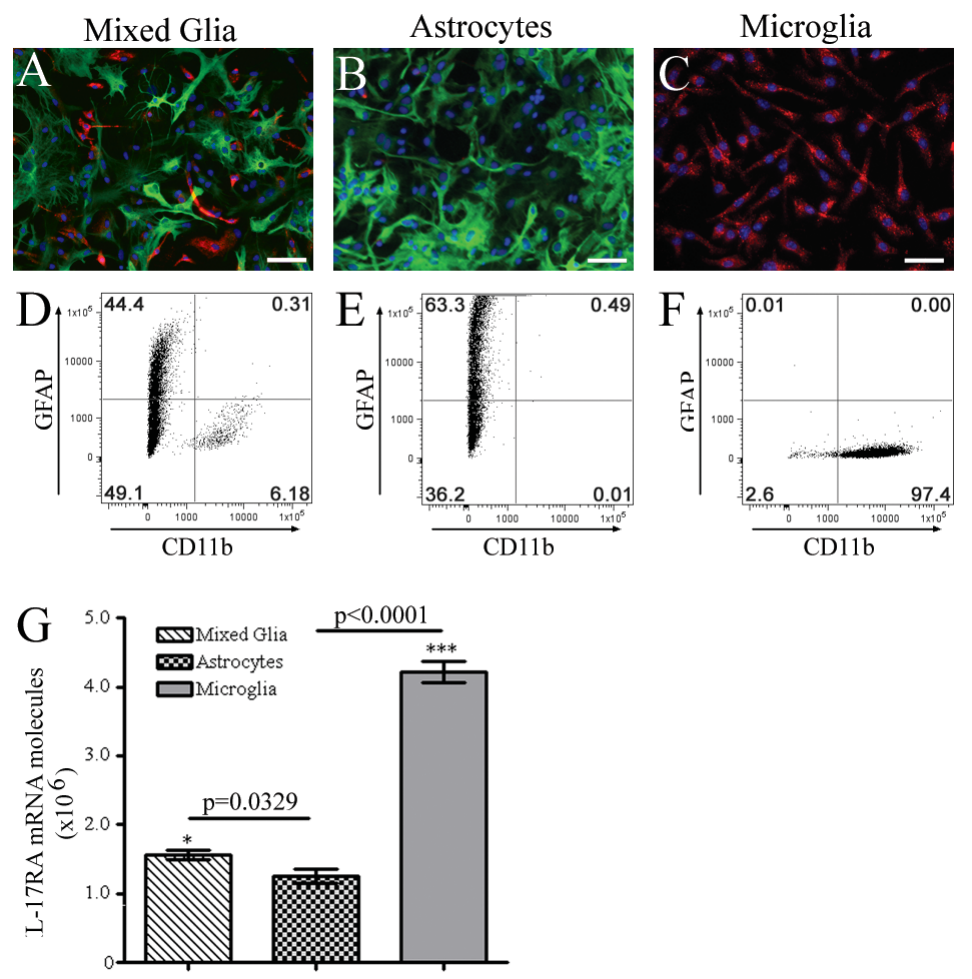
**IL-17RA expression in astrocytes and microglia *in vitro***. (A-C) *Phenotypic characterization of glial cells by immunofluorescence*. Mixed glial cultures (A), and purified astrocytic (B) and microglial cultures (C) were established from neonatal C57BL/6 mice. Cultures were stained with anti-GFAP antibody (astrocytic marker; green) and anti-CD11b (microglial marker; red) and counterstained with nuclear stain DAPI (blue). Mixed glial cultures primarily consist of astrocytes (70–80%) and microglia (5–10%); whereas, purified astrocyte cultures consist of 80–90% GFAP-positive cells. Purified microglial cultures are 98–99% CD11b-positive. (D-F). *Flow cytometry*. Glial cells were immunostained for flow cytometric analysis. Mixed glial cultures (D) contain both GFAP- and CD11b-positive cells. Astrocyte cultures were free from microglia (< 0.5%) (E) and microglial cultures free of astrocytes (< 0.5%) (F). (G) *IL-17RA expression in vitro*. mRNA was extracted from glial cultures and IL-17RA expression was quantified by RT-PCR using a primer set from exon boundary 1–2. Data represent the mean ± SEM expression of total IL-17RA mRNA from isolated cultures from three different batches of donors. IL-17RA is expressed 4-fold higher in microglia compared to astrocytes (***p < 0.0001). Mixed glial culture confers more expression of IL-17RA mRNA in comparison to astrocyte cultures devoid of microglia (*p = 0.0329).

To determine whether distinct CNS cell types express IL-17RA, we used *in vitro *cell cultures. This averted technical difficulties of high background staining levels *in vivo *in the CNS, and also importantly eliminated the influence of infiltrating peripheral immune cells that occurs in EAE. We specifically examined glial cell cultures because astrocytes and microglia play significant roles in the development of both innate and adaptive immune responses in the CNS [34]. Using day 0 neonatal CNS tissue we first established mixed glial cultures containing both astrocytes and microglia (Figure 5A). Enriched sub-cultures were then established with astrocytes free of microglia, or microglia free of astrocytes (Figure 5B and 5C respectively). We verified isolated culture purities by flow cytometry (Figure 5D–F) and found that microglial cultures were 98–99% pure. Astrocyte cultures were 60–80% GFAP-positive by flow cytometry, more than 85% pure by immunofluorescence, and devoid of CD11b positive cells. RNA was extracted from glial cultures (mixed glia, microglia or astrocytes); cDNA was synthesized and analyzed by RT-PCR using probes from exon boundary 1–2. IL-17RA was expressed in all glial culture systems with highest expression in microglial cultures (Figure 5G). For comparison, IL-17RA expression was measured by RT-PCR in cultures enriched for oligodendrocytes and was almost 50% lower than in purified astrocytic cultures (data not shown), likely reflecting the presence of 60% astrocytes in these enriched cultured.

**Figure 6:**
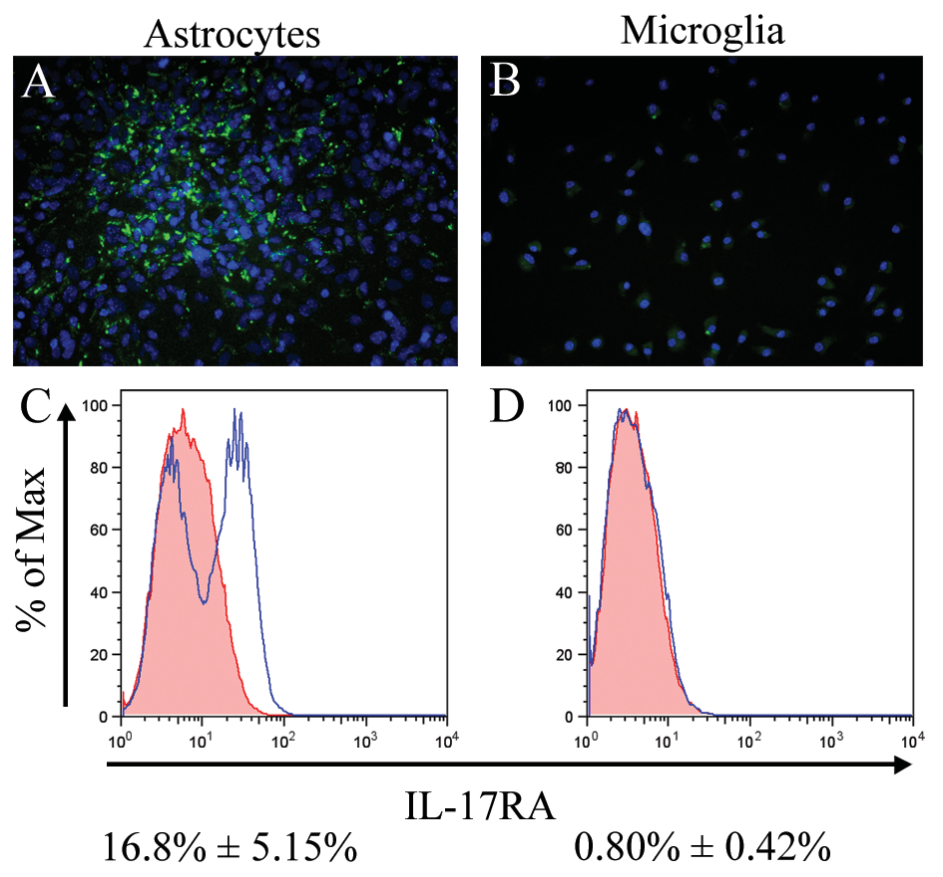
**Detection of IL-17RA protein on the cell surface of neonatal glial cells**. Purified astrocytes (A) and microglia (B) cultured on chamber slides were fluorescently labeled with anti-IL-17RA antibody and CY2 -conjugated Hamster anti-goat IgG secondary antibody. The majority of astrocytes showed punctuate surface staining (A), whereas IL-17RA staining was much weaker in microglia (B). Binding of polyclonal-anti-mouse IL-17R-fluorescein on the surface of isolated astrocytes and microglia was tested using flow cytometric analysis. Cells were double labeled with anti-GFAP (intracellular marker for astrocytes) and IL-17RA antibody (C) or anti-CD11b (for microglia) and IL-17RA antibody (D). Protein G- coupled normal goat-IgG conjugated with carboxyfluorescein was used as isotype. Cells were gated either for GFAP or for CD11b, and IL-17RA expression on gated cells shown in single parameter FACS plot against the isotype staining demonstrates a prominent population of IL-17RA positive astrocytes, but only rare positive microglia.

To determine whether neonatal glial cells in culture also express IL-17RA at the protein level we labeled purified astrocytes and microglia with commercially available anti-IL-17R antibody raised against a peptide mapping at the N-terminus of IL-17RA. We observed a significant amount of punctuate staining for IL-17RA at the cell surface of astrocytes (Figure 6A) whereas, in microglia, IL-17RA staining was much weaker (Figure 6B). To quantitatively determine the expression of IL-17RA protein on our neonatal purified astrocytes and microglia we stained the cells with polyclonal anti-mouse IL-17R-fluorescent antibody which was designed to determine the density of IL17RA on cell surface by flow cytometry. Protein G-purified normal carboxy fluorescent -conjugated goat IgG was used as isotype control. Flow cytometric analysis of IL-17RA- fluorescent antibody staining demonstrated that 16.8 ± 5.15% of GFAP positive cells were positive for IL-17RA (Figure 6C) expression where as only 0.80 ± 0.42% of cells were positive for IL-17RA in the purified microglial population (Figure 6D). Both immunofluorescence data and flow cytometric data are in agreement that glial cells express IL-17RA at the protein level and among the glial cells astrocytes express far more IL-17RA than microglia.

### Glial cells transduce IL-17A signal in vitro

**Figure 7:**
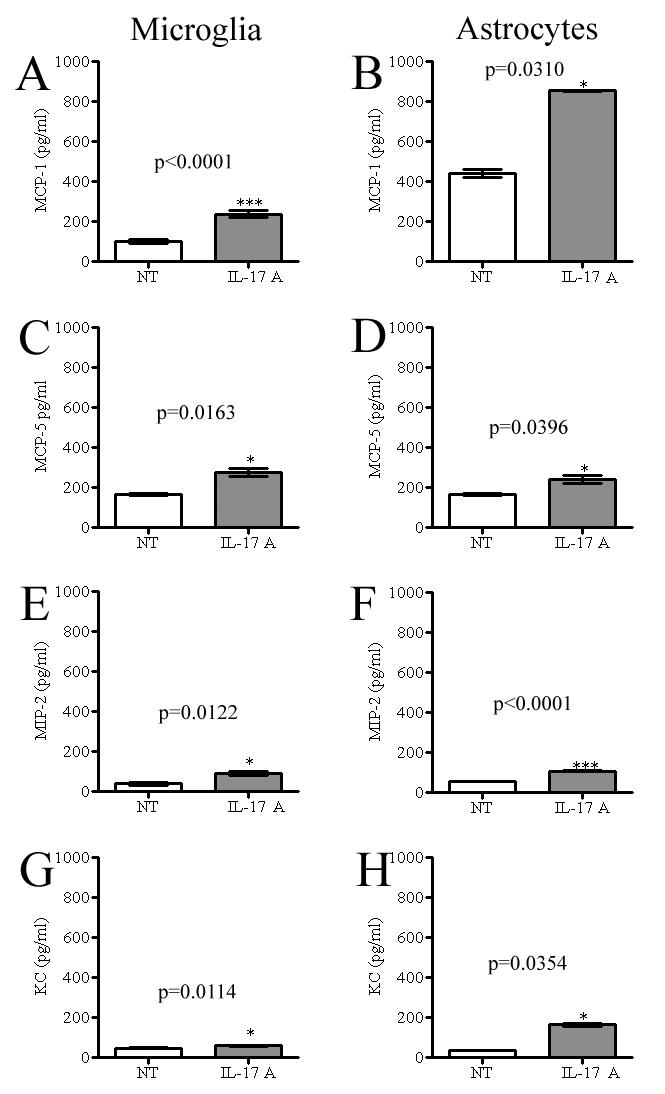
**Exogenous IL-17A treatment induces chemokine secretion *in vitro***. (A-H) Isolated astrocyte and microglia cultures were treated with IL-17A (10 ng/ml). Culture supernatants from treated and non-treated cultures were collected at 12 hr and assessed for chemokine levels by a multiplex array system. In response to IL-17A, microglia and astrocytes each upregulated secretion of MCP-1, MCP-5, MIP-2 and KC. One experiment of three is shown. *p < 0.05, ***p <0.0001. Mean ± SEM was generated from three multiple wells of the single experiment.

To study the functional responsiveness of glial cells to IL-17A without the complex influence of inflammatory networks present during pathogenesis, we treated glial cultures with exogenous IL-17A. Using a multiplex array system we examined secretion of 29 different analytes including cytokines, chemokines, matrix metalloproteinases and growth factors (Table 1) by glial cells cultured for 12 hr in the presence or absence of exogenous IL-17A (10 ng/ml). Microglia and astrocytes each constitutively expressed several chemokines (MCP-1, MCP-5, MIP-2, MIP-1α, MIP-3β, KC and RANTES) (data not shown). IL-17A treatment significantly upregulated the expression of MCP-1, MCP-5, MIP-2 and KC (Figure 7A–H), with MIP-2 and KC near the lower limits of detection. MIP-1α, MIP-3β and RANTES expression were not significantly affected by IL-17A in either astrocyte or microglia cultures, and no significant cytokine upregulation was observed either (data not shown). While TGFβ and MMPs were constitutively expressed by astrocytes and microglia, exogenous IL-17A treatment did not significantly alter this expression (data not shown). We also treated cells with IL-17A at a concentration range of 1–100 ng/ml and examined the secretion of analytes (Table 1) at various time points. We observed maximal upregulation of chemokines when IL-17A was used at 10 ng/ml at the 12 hr time point, with no difference between 10 and 100 ng/ml IL-17A treatments from 12 to 48 hr (data not shown).

### Exogenous IL-17A does not alter IL-17RA expression in glial cultures

**Figure 8:**
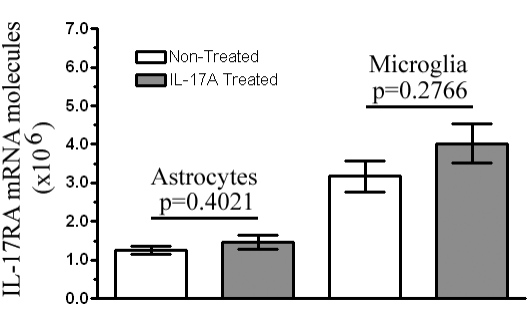
**Exogenous treatment of IL-17A does not alter IL-17RA expression in glial culture**. mRNA was isolated from either non-treated resting culture or IL-17A (10 ng/ml) treated culture supernatants at 12 hr *in vitro*. IL-17RA gene expression was measured by RT-PCR using a primer set from exon boundary 1–2. Data represent the mean expression from three different non- treated and IL-17A- treated culture batches ± SEM. IL-17A treatment did not alter IL-17RA expression in neonatal glial cells (*p > 0.05).

To ensure that changes in chemokine expression induced by IL-17A were due to signaling through constitutively expressed IL-17RA, as opposed to an increase of IL-17RA expression, we evaluated the influence of IL-17A treatment on IL-17RA expression in astrocytes and microglia. IL-17A did not significantly alter the constitutive expression of IL-17RA mRNA (p > 0.05) (Figure 8). This infers that upregulation of chemokines by glial cells was due to exogenous IL-17A signaling through constitutively expressed IL-17RA.

## Discussion

Increasing evidence suggests that IL-17A and Th17 cells play a major role in autoimmune inflammation, but there are gaps in our understanding of IL-17RA signaling mechanisms. IL-17RA is expressed in most tissues examined to date and activates many of the same signaling cascades as innate cytokines such as TNFα and IL-1β [35,36]. Thus IL-17A is considered an important bridging molecule between the adaptive and innate immune systems [15,37]. Furthermore, emerging knowledge regarding IL-17A/IL-17RA signaling in numerous tissues suggests a broader role in health and disease beyond the immune system. Given this importance of IL-17RA signaling, it is of particular interest to understand the role of IL17RA signaling in the CNS of mice with autoimmune inflammatory disease.

In our present study we demonstrated that healthy mouse CNS constitutively expresses IL-17RA. To investigate cell-specific expression of IL-17RA in healthy mouse CNS *in vivo *we performed immunofluorescence on brain, spinal cord and spleen tissue sections. Spleen cells had detectable Il-17RA expression both at mRNA and protein levels. While our detection of IL-17RA mRNA in whole CNS tissues by RT-PCR suggests that IL-17RA is expressed, unfortunately immunohistochemical staining in the CNS was not interpretable, possibly due to the quality of available antibodies, or perhaps due to the overall expression levels. Indeed, in human studies, Kebir et al. also were unable to detect IL-17RA expression *in situ *in healthy CNS [27]. They did demonstrate, however, that IL-17RA is expressed on CNS endothelial cells in MS lesions.

We also observed that, in comparison to healthy mice, the expression of IL-17RA is significantly increased in the CNS of mice with EAE. This is of particular relevance to MS and EAE as Th17 cells and IL-17A have been implicated in disease pathogenesis [3,5]. As the CNS in the EAE model contains peripheral immune cells that have infiltrated during the inflammatory process, it is likely that increased expression of IL-17RA is partly due to the abundance of these cells, but increased IL-17RA expression may also be due to increased expression in resident CNS cells. Our *in vitro *results suggest that expression can occur in astrocytes and microglia, although similar low level expression in neurons has not been excluded. In either case, whether from resident cells or infiltrating cells, increased IL-17RA expression in the inflamed CNS suggests a heightened responsiveness to IL-17A signaling. Indeed, we observed significant constitutive expression of the IL-17RA mRNA in both astrocytes and microglia in purified cultures, free of peripheral immune cells. At the protein level we observed that mainly astrocytes express IL-17RA, with microglia expressing only very little. The differential expression of mRNA and protein in microglia and astrocytes could be due to a combination of gene expression regulatory mechanisms and differences in post translational control. An important question is whether this level of expression has any functional significance, and our functional studies *in vitro *suggest that it may.

Although produced primarily by T cells, IL-17A is known to trigger a variety of target cells to secrete inflammatory mediators, including chemokines, cytokines and cell surface receptors [28]. We verified that IL-17RA expression on glial cells is functional by treating these cultures with exogenous IL-17A and examining the expression of a range of targets serving as surrogate markers of IL-17RA signaling. We chose not to activate these cultures with bacterial products or potent endogenous activators of inflammation (such as TNF-α or IFN-gamma) so as not to obscure the constitutive profile of IL-17RA expression and function in glial cells. Our functional studies demonstrate that IL-17A treatment significantly upregulated the expression of MCP-1, MCP-5, MIP-2 and KC in both purified astrocyte and microglia cultures. This suggests that expression in even a small subpopulation of cells as seen in microglial cultures is sufficient to mediate a significant functional response. Moreover, upregulation of chemokines by glial cells was exclusively due to exogenous IL-17A signaling through constitutively expressed IL-17RA, as exogenous IL-17A treatment did not significantly alter the expression level of IL-17RA mRNA in microglia or astrocyte cultures.

These results suggest IL-17A may exert some of its proinflammatory effect through direct interaction with its receptor on glial cells to regulate expression of several chemokines. Some of these chemokines are known to play a role in amplifying the inflammatory reaction in EAE/MS [38]. Moreover, the upregulation of these chemokines suggests that the low level of IL-17RA expressed on astrocytes and microglia is functional, and may have biological significance in CNS inflammation. In mice with EAE, IL-17A may be secreted from CD4+ T cells/Th17 infiltrating cells and bind to IL-17RA on CNS resident glial cells, which in turn can secrete chemokines that attract a range of other inflammatory cells, such as KC and MIP-2 that are known to recruit neutrophils [39]. Moreover, glial cells may be part of the cellular machinery that IL-17A uses in the CNS to steer local inflammation. Interestingly, IL17 may lead to production of a different set of inflammatory mediators in other cell types, as IL-17 binding to an IL-17 receptor expressed on epithelial, endothelial, and fibroblastic stromal cells results in the secretion of IL-1, TNF-alpha, IL-6, IL-8, or prostaglandin E2 [40]. Together with our results, this suggests that IL-17 binding may trigger cell-type-specific responses that require further characterization in specific disease models.

## Conclusion

Together, our studies demonstrate that both astrocytes and microglia are responsive to IL-17A. However, full functional stimulation by IL-17A may require additional inflammatory signals (e.g. IFN-γ, TNF-α, IL-1β, LPS) not present in our *in vitro *system. Indeed, the cellular response elicited in glial cells by IL-17A will likely differ depending on the inflammatory status of the tissue. In addition, cross communication between IL-17A and other cytokine signaling systems would likely modify the response of glial cells to IL-17A. Infiltration of IL-17A-secreting T cells has clearly been demonstrated to be a pathogenic event in EAE. The resultant cellular and chemokine milieu and its effect on IL-17RA signaling in glial cells warrant detailed study in the future. Nonetheless, here we have demonstrated for the first time that IL-17RA is expressed constitutively in mouse CNS, is upregulated during EAE, and is expressed on astrocytes and microglia suggesting a role for glial IL-17A signaling in mediating CNS inflammation.

## Competing interests

The authors declare that they have no competing interests.

## Authors' contributions

JDS led all aspects of this work including experimental design, participated in or supervised all experimental procedures, analyzed and interpreted data, and drafted the manuscript. BC made substantial contributions to experimental conception and design, and was involved in critical revisions of the manuscript. RM performed glial cell isolation and RT-PCR data analysis. SS conducted RNA extraction, IL-17RA cloning, sequence analysis, and RT-PCR experiments. MLC performed IL-17RA protein expression studies. JS participated in cloning of IL-17RA and sequence analysis. DCF participated in data interpretation, and critical revisions of the manuscript. KSS participated in study design, data interpretation, and preparation and critical revisions of the manuscript. AMR participated in study design and interpretation.
